# Cuproptosis as a therapeutic target in cancer: a Systematic Review and bibliometric analysis of the research landscape

**DOI:** 10.3389/fonc.2025.1566986

**Published:** 2025-05-16

**Authors:** Chuhan Jiang, Huizhen Xin, Yuhang Liu, Yangyang Han

**Affiliations:** ^1^ Department of Biology, School of Basic Medical Sciences Xinjiang Medical University, Urumqi, China; ^2^ Xinjiang Key Laboratory of Molecular Biology for Endemic Diseases, Xinjiang Medical University, Urumqi, China

**Keywords:** cuproptosis, cell death, cancer, Citespace, bibliometric analysis

## Abstract

**Background:**

Cuproptosis is a new form of cell death induced by intracellular copper overload. With the deepening of research, the research of cuproptosis in the field of cancer has become a hot topic. The bibliometric analysis of cuproptosis research can provide valuable insights into the development of this field.

**Method:**

In this study, the Web of Science Core Collection database was used to obtain literature, and the screened data were imported into CiteSpace software for analysis. We use this data for visualization analysis and made knowledge maps including authors, countries, institutions, journals, and keywords.

**Results:**

1140 literature was obtained from Web of Science from 2001 to 2024. The results indicate a consistent upward trend in the number of publications in this field. Moreover, a particularly significant surge in the frequency of citations has been observed since 2022. Through a systematic analysis, we found that in the current field of cancer research on cuproptosis, breast cancer, lung cancer, hepatocellular carcinoma and colorectal cancer have more research results.

**Conclusion:**

This article describes how copper ions regulate cell death, particularly in cancer therapy, and requires an in-depth understanding of the complexity of copper metabolism and its specific mechanisms of action in cell death. The work provides a panoramic view of the research landscape on cuproptosis in cancer, highlighting its potential as a therapeutic target and the need for further exploration into its mechanisms and clinical applications. With the depth of research, it is expected that cuproptosis will continue to be a hotspot in cancer treatment research. In addition, it provides a solid theoretical foundation and experimental basis for the development of new anti-tumor therapeutic strategies.

## 1 Introduction

Copper (Cu), one of the trace metals essential for life (1), is delicately regulated in the body - too little copper can interfere with the basic function of copper-binding enzymes, while too much copper can overwhelm or even kill cells. The precise mechanisms and forms of copper-induced cell death have remained unclear for an extended period.

The phenomenon of copper ion-induced cell death has its origins in the 1980s; however, the underlying mechanisms that govern this process remain elusive. In 1989, the observation that “excess copper induces cell death” was first reported (2). By 2006, clinical studies had confirmed the presence of abnormally elevated copper concentrations in tumor tissues (3). However, initial interpretations attributed this phenomenon solely to ROS accumulation or non-apoptotic cell death. The term “cuproptosis” was coined to describe the process of cell death induced by copper and oxidative stress, which is distinct from “ferroptosis,” another form of cell death involving metal ion toxicity and oxidative stress. Initially, the similarity in the inhibitors of these two processes (e.g., ROS scavengers) led to the misclassification of cuproptosis as a ferroptosis subtype. In 2008, the hypothesis that “copper-induced cell death may not follow apoptotic pathways” was proposed (4). Subsequent studies in 2016 demonstrated that DSF suppressed tumor growth via ROS-dependent mechanisms, suggesting a death pathway independent of apoptosis (5). However, the absence of molecular evidence led to the relegation of these findings to the broader category of non-apoptotic cell death. A seminal study in 2017 revealed that copper inhibits Npl4, thereby disrupting proteasome function and implicating proteotoxic stress as a key pathway (6). In 2019, researchers from the Broad Institute of MIT and Harvard, led by Todd Golub, identified two small-molecule Cu^2+^ ionophores, disulfiram (DSF) and elesclomol (ES), which selectively transport copper ions across cell membranes (7). These agents were shown to selectively kill specific drug-resistant cancer cells; however, their precise mechanisms of action remained to be elucidated at the time. Until 2022, a study conducted by Tsvetkov and colleagues was published in the journal Science. They found that excess intracellular copper leads to proteotoxic stress aggregation of lipoylated dihydrolipoamide S-acetyltransferase (DLAT), which is associated with the mitochondrial tricarboxylic acid (TCA) cycle. This leads to and a new way of cell death called cuproptosis (8). Cuproptosis is a recently identified novel form of cell death that has been characterized as a mitochondria-dependent pathway that is triggered by intracellular copper ion overload. This novel form of cell death is distinct from classical death mechanisms such as apoptosis and necroptosis. The hallmarks of cuproptosis are primarily characterized by mitochondrial stress caused by the aggregation of lipoylated mitochondrial enzymes and the loss of Fe-S cluster proteins. This process disrupts mitochondrial metabolic homeostasis.

Cancer is still one of the primary causes of death worldwide. Researchers are continuously developing and exploring various synthetic anticancer compounds (9). What’s more, a growing number of studies are concentrating on using cuproptosis-related genes (CRGs) to establish the association between cuproptosis and manifold types of cancers, including breast cancer, colorectal cancer, prostate cancer, bladder cancer, lung cancer, and endometrial cancer (10–15). Copper plays a dual role in carcinogenesis: disruption of its homeostasis can both drive tumorigenesis and induce cell death. Within the tumor microenvironment, copper promotes malignant transformation through multiple mechanisms. For instance, copper activates the MEK1/ERK signaling pathway by directly binding to the H188/M230 sites on MEK1, thereby enhancing the proliferation of BRAF-mutant tumors (16). Copper interacts with key molecules across various signaling pathways to regulate tumor growth. Specifically, it activates downstream effectors by acting on different molecules in the PI3K-AKT signaling pathway, thereby promoting tumor cell proliferation (17). Angiogenesis is closely associated with tumor development. Copper can stimulate endothelial cell proliferation and promote vascular hyperplasia by upregulating critical angiogenic factors such as interleukins and vascular endothelial growth factor (VEGF) (18). Notably, interleukins facilitate cellular copper uptake through STEAP4 (six-segment transmembrane epithelial antigen of prostate 4), which activates E3 ligases, enhances NF-κB (nuclear factor-κB) activity, and suppresses Caspase 3 function, ultimately promoting tumor cell growth (19).

In recent years, numerous researchers have studied the important link between cuproptosis and cancer. This suggests that an increase in copper concentration in the cell can be used for the purpose of selectively killing cancer cells (20). In addition, cuproptosis has become a popular research topic in cancer treatment and prognosis, and the related literature has surged. This study employed a systematic approach to collate and analyze the existing literature on cuproptosis in cancer. Research trends and hotspots between 2001 and April 29, 2024 were explored through CiteSpace software and bibliometric analysis. These findings set the stage for further research into cuproptosis in cancer and raise the possibility of anti-tumor therapies.

This study aims to comprehensively map the knowledge landscape and developmental trends of cuproptosis in cancer research through systematic literature review and bibliometric analysis. By utilizing CiteSpace software to construct author collaboration networks, country/institution distributions, journal contributions, and keyword co-occurrence maps, the research reveals core research forces, knowledge evolution trajectories, and cutting-edge hotspots in this emerging field. Through analyzing annual publication trends, high-impact literature, and burst keywords, it identifies breast cancer, lung cancer, liver cancer, and colorectal cancer as current focal malignancies in cuproptosis research. Furthermore, the study systematically examines the molecular mechanisms of cuproptosis, its cancer-type specificity, and therapeutic applications in oncology through comprehensive review.

## 2 Method

### 2.1 Data collection

Given that the Web of Science Core Collection (WoSCC) is a frequently utilized source database in bibliometric analysis, it was selected for the purpose of data retrieval. The search formula was TS= “cuproptosis” OR “cell death via copper “ OR “copper induces cell death” AND TS= “cancer” OR “tumor “ OR “pan-cancer” OR “neoplasm” OR “carcinoma” OR “lymphoma” OR “leukemia” OR “sarcoma” OR “adenocarcinoma” OR “osteosarcoma” OR “melanoma” OR “oncology” OR “malignancy”. The search date was from inception to April 29th,2024. A total of 2229 articles were retrieved.

### 2.2 Data analysis and visualization

**Figure 1:**
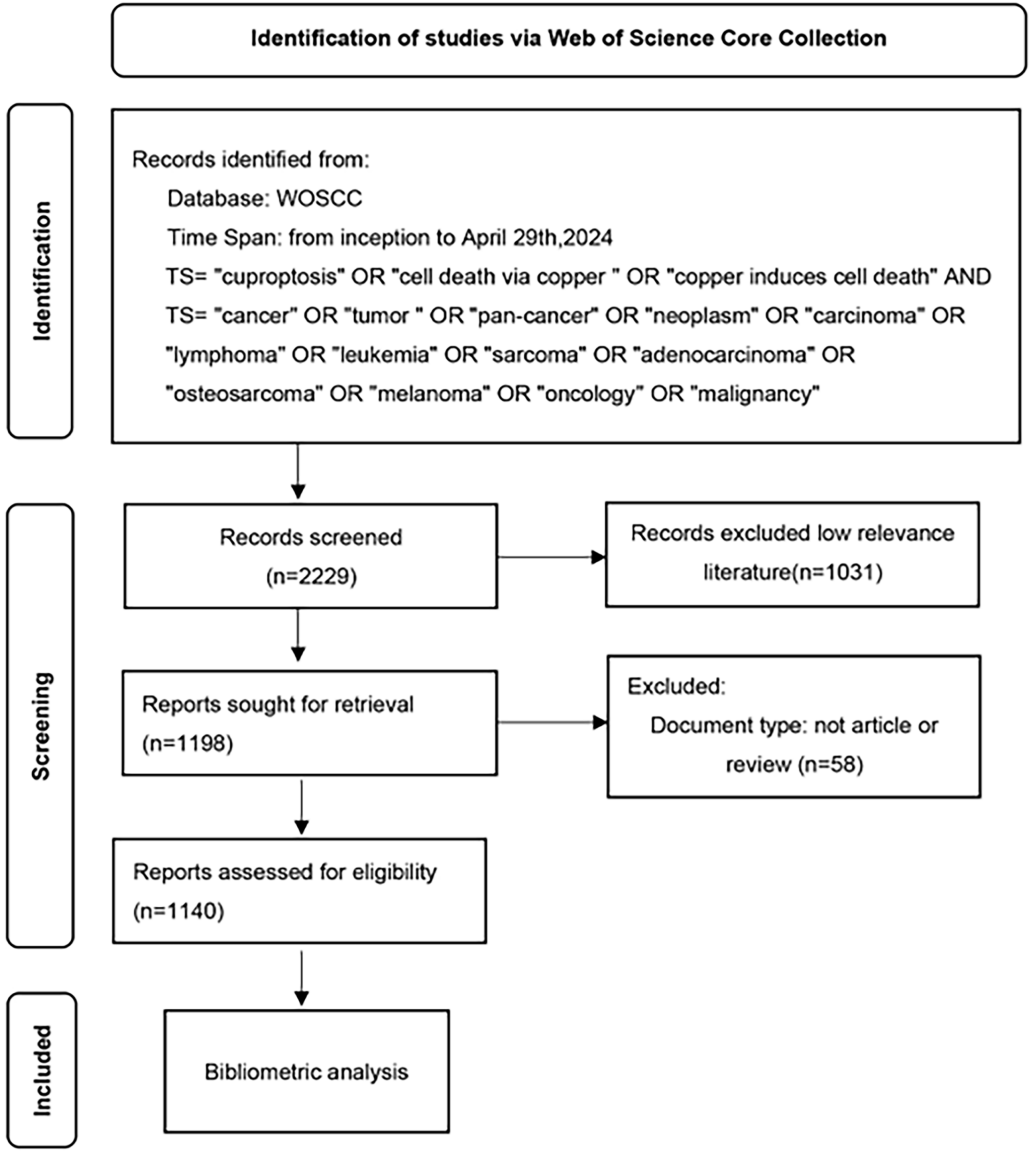
A study flow diagram of Cuproptosis in cancers from WoSCC. WoSCC, web of science core collection.

Document types are limited to articles and opinion pieces and all other document types were excluded. Two researchers screened the literature, 1031 papers with low relevance excluded based on their title, abstract and keywords. In the end, 1140 high quality documents with high-relevance were obtained. 1,077 articles and 63 reviews are included. These papers have been downloaded with the record contents of “Full Record and Cited References” and “Plain Text” file formats. Then renamed all the files to “download_xxx.txt”. **Figure 1** shows the flow diagram of the study.

CiteSpace is a software program designed for the visualization of information. This software was led by Dr. Chaomei Chen, and it is constructed upon the Java programming language and the theory of citation analysis. We used CiteSpace (version 6.3.R1) for visualizing knowledge mapping to visually summarize the research progress and research hotspots of cuproptosis in cancer and to predict its subsequent research trends. The analysis of publication trends was conducted using Microsoft Office Excel 2021 software. Co-occurrence analysis of authors, countries, institutions, journals, and keywords were made. Furthermore, a clustering and emergence analysis of 1,140 literature sources was carried out. The literature visualization analysis displayed all nodes as citation tree-rings. The connecting lines between the nodes represented associations, with thicker lines indicating stronger associations. Node size represented frequency, and node color represented different clusters. Betweenness centrality measures the importance of a node in a graph. Nodes with centrality not less than 0.10 are highlighted with a purple ring, with thicker rings indicating higher centrality. In the domain of bibliometric analysis, betweenness centrality emerges as a pivotal metric, with the capacity to quantify the frequency with which a node lies on the shortest paths between other node pairs within a network. This measure unveils a node’s potential to regulate information flow by serving as a “bridge” or intermediary. Its significance lies in identifying cross-domain pivotal documents or authors, elucidating structural holes within knowledge networks, and mapping evolutionary trends. It offers a framework for enhancing the efficacy of collaborative networks and monitoring the evolution of research domains.

## 3 Results

### 3.1 Analysis of annual publication

**Figure 2:**
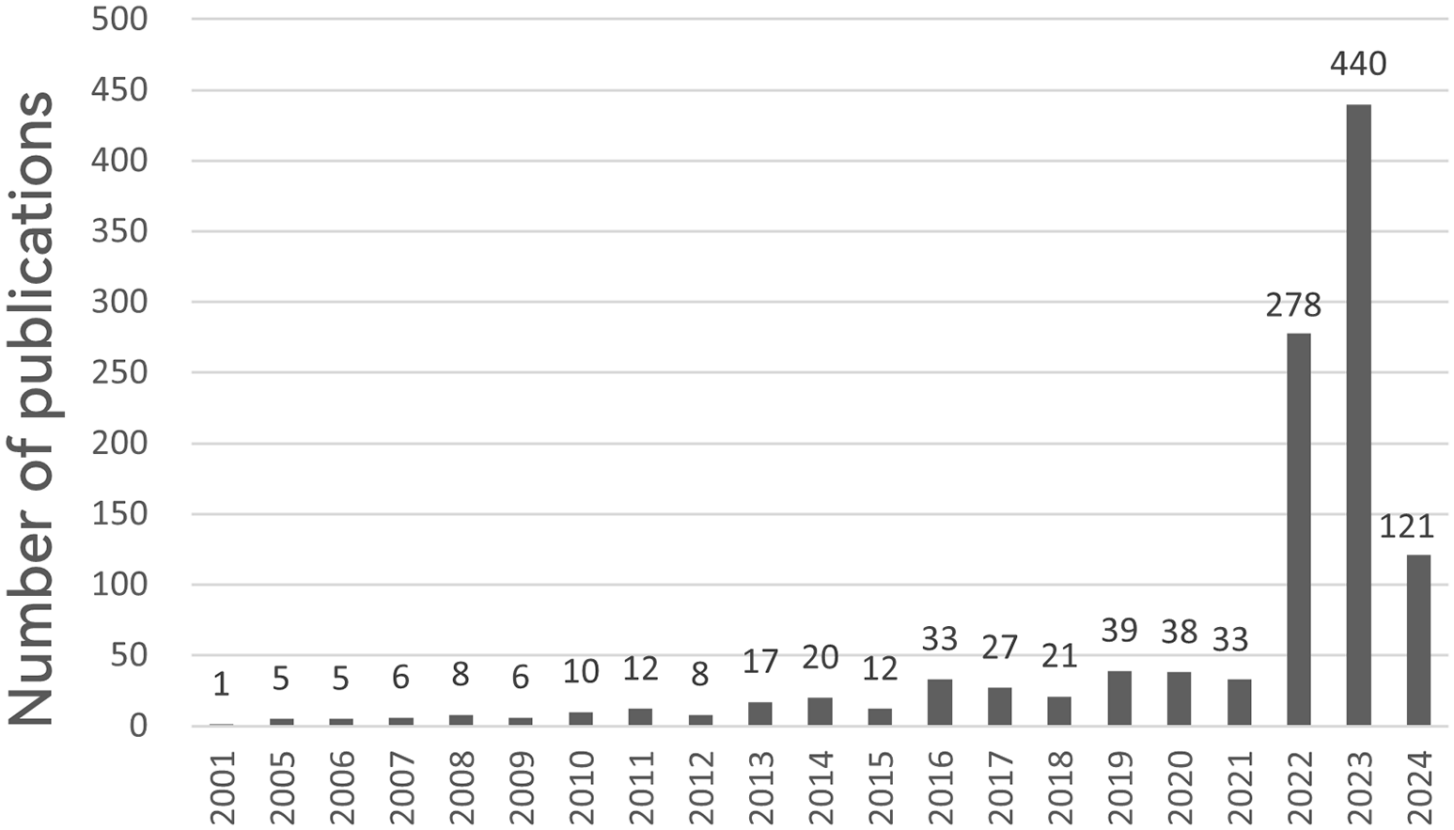
Annual number of publications on cuproptosis in cancer treatment, 2001-2024. The horizontal coordinates represent the year of publications and the vertical coordinates indicate the number of publications.

We explored changes in cuproptosis in cancer research over time by quantifying the number of published papers. An analysis of fluctuations in the annual publication volume can serve as a valuable indicator of emerging research trends. From inception to 2024, 1140 publications were included in this study. The number of publications per year is presented in **Figure 2**. As the study only covers up to April 2024 and the data for 2024 is incomplete (n=121). A stable trend in publication output was observed over the period 2001-2021, whereas cuproptosis in cancer research exploded and increased yearly from 2022 (n=278) to 2023 (n=440), making it a hot topic of research. It is expected that the volume of published literature will continue to increase in 2024 and that research in the areas of cuproptosis in cancer will intensify worldwide.

### 3.2 Analysis of authors, countries and institutions

**Figure 3:**
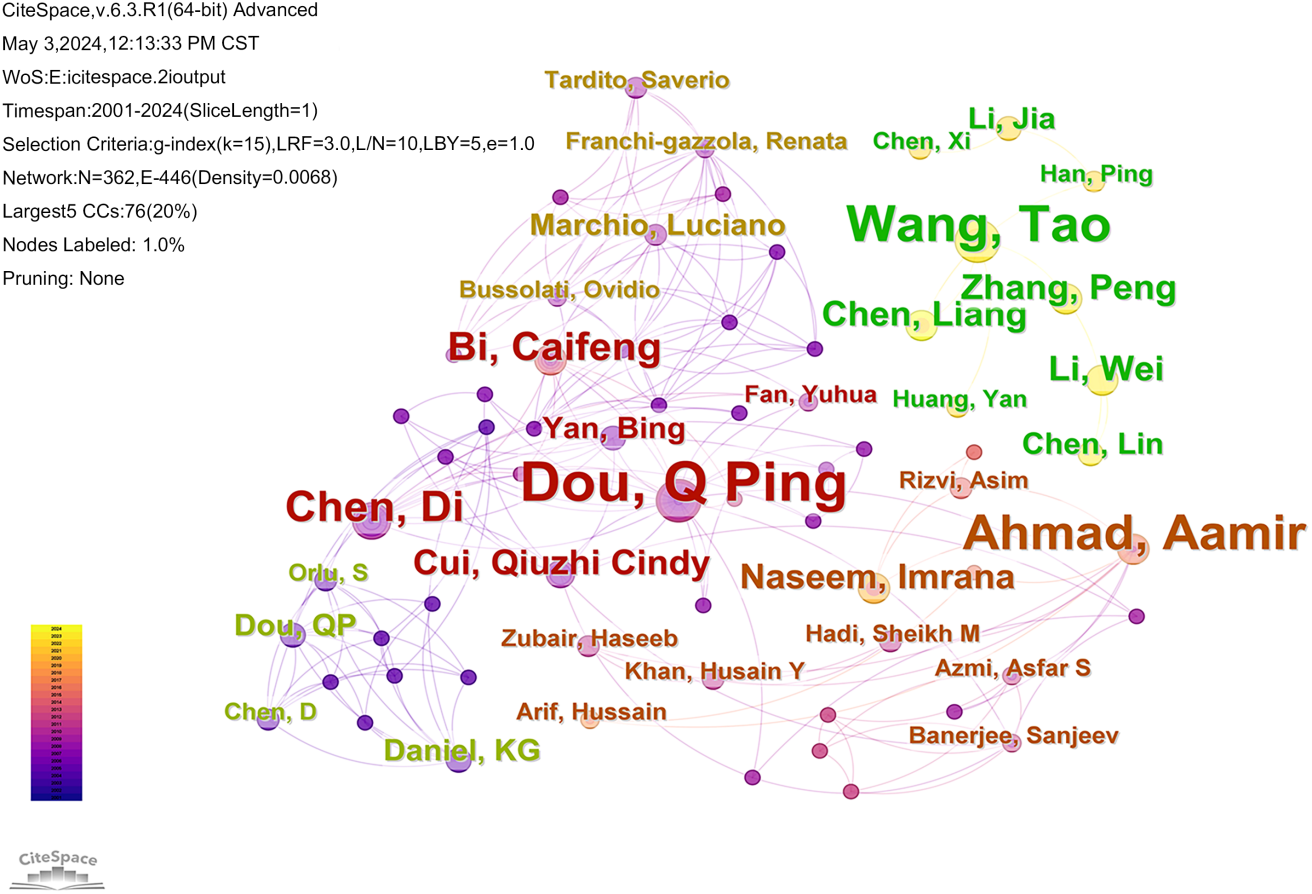
The map illustrates the authors who have contributed to the published literature on cuproptosis in cancer. The nodes in the graph represent the authors, nodes with the same color and connections indicate that there is collaboration between the authors.

The analysis could reflect the authors who are active in cuprotosis in cancer research. The CiteSpace software retrieved 1140 documents from 6834 authors, and the author network is mapped in **Figure 3**. In the top 10 authors in terms of publication numbers, 3 scholars have ≥8 publications, namely Dou, Q Ping (n=12), Wang, Tao (n=9), and Ahmad, Aamir (n=8). In terms of total citations, Tsvetkov P (n=690) is ranked as the top author, and analyzed with the data of h-index, it’s evident that the papers of this scholar have great academic influence and are widely recognized in the field of cuproptosis.

**Figure 4:**
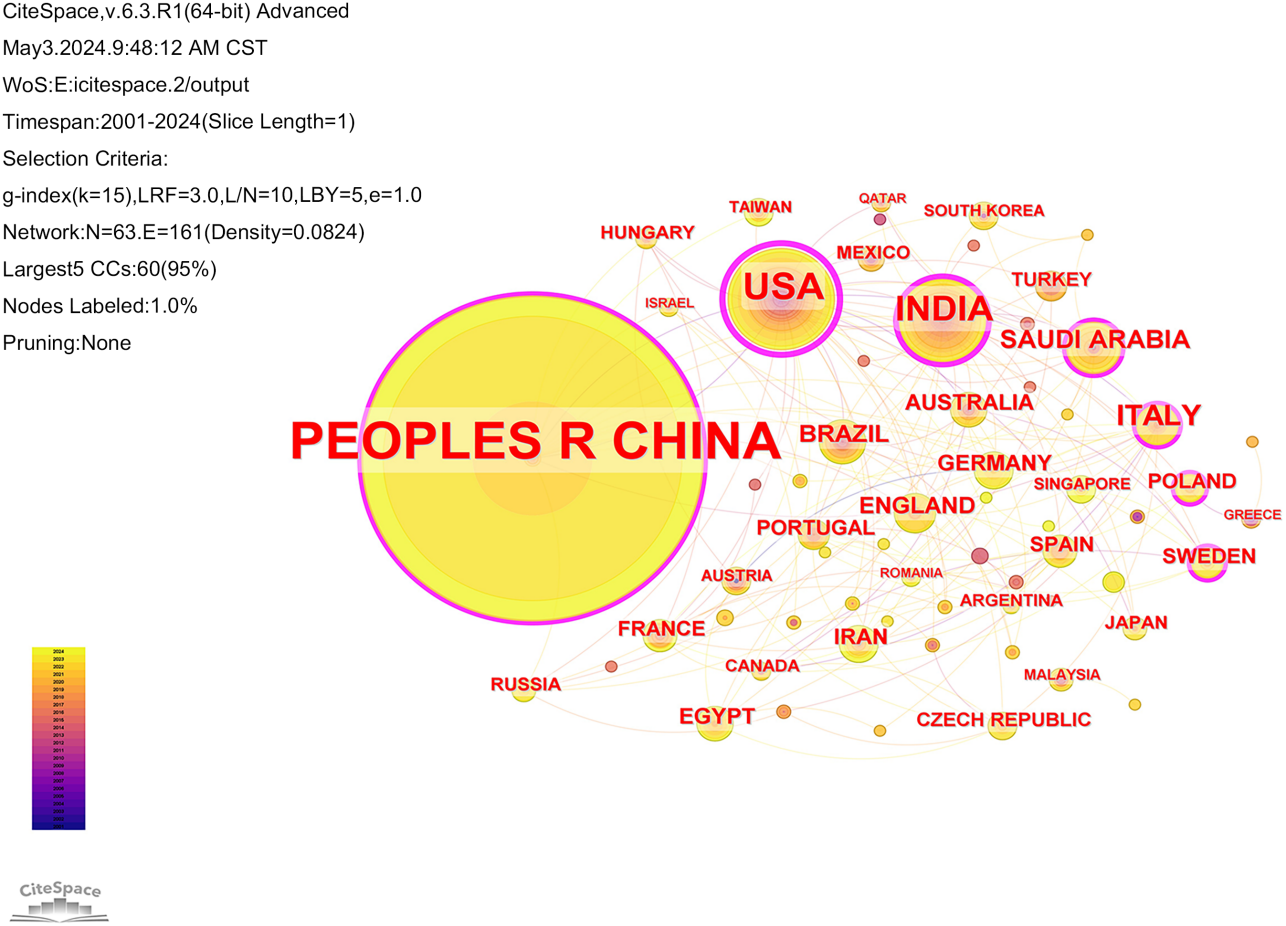
Collaboration analysis of countries. Node size represented frequency and nodes with centrality not less than 0.10 are highlighted with a purple ring.

**Figure 5:**
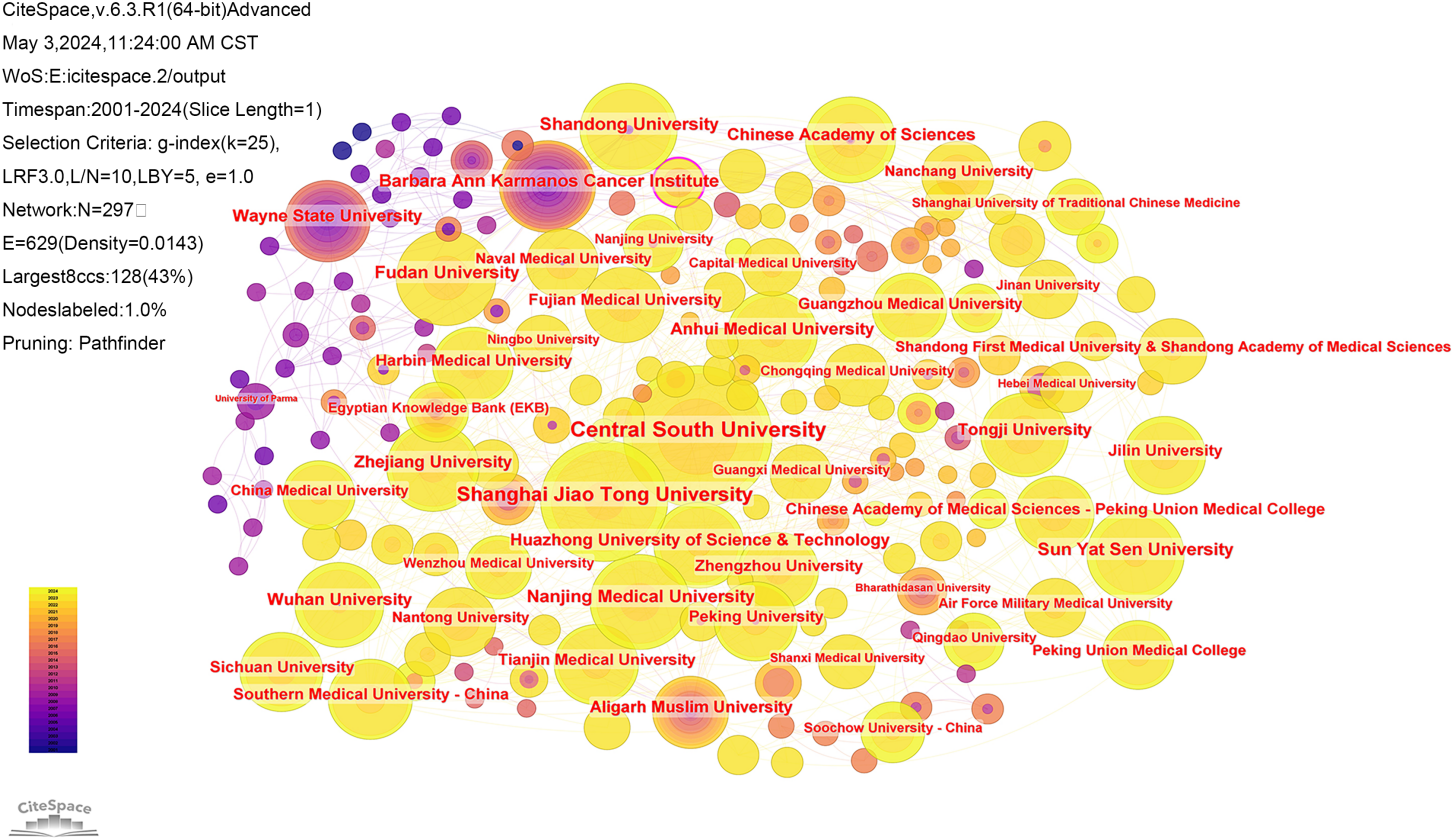
Collaboration analysis of institutions.

Of the literature included in the analysis, 3724 institutions from 63 countries have contributed papers in this field. The collaborative network of countries and institutions was mapped using CiteSpace software (**Figures 4**, **5**). Combined with the h-index data to analyze the images, it becomes apparent that despite only accounting for 10% of the included literature, the United States has a high centrality of 0.62. As the country with the highest number of articles published in the field (71.4%), China should strengthen international cooperation and promote research in the area of cuproptosis in cancer to a more in-depth level. **Tables 1** and **2** display the top 10 countries and institutions based on the number of publications. Furthermore, eight of the top ten institutions were Chinese universities, with Central South University having the highest number of papers (n=62). The other two were American universities, Barbara Ann Karmanos Cancer Institute (n=27) and Wayne State University (n=24). The high centrality of Chinese Academy of Sciences (CAS) indicates that CAS has an important reference value in the literature on cuproptosis in cancer.

**Table 1:** Top 10 prolific countries publishing research in the field of cuproptosis in cancer.

Ranking	Count	Centrality	Countries	Percentage(n/1140)
1	814	0.11	PEOPLES R CHINA	71.40%
2	114	0.62	USA	10.00%
3	78	0.27	INDIA	6.84%
4	34	0.17	ITALY	2.98%
5	22	0.12	SAUDI ARABIA	1.93%
6	19	0.06	BRAZIL	1.67%
7	16	0.03	ENGLAND	1.40%
8	14	0.05	GERMANY	1.23%
9	14	0.04	AUSTRALIA	1.23%
10	14	0.02	IRAN	1.23%

**Table 2:** Top 10 productive institutions conducting cuproptosis in cancer research.

Ranking	Count	Centrality	Institutions	Percentage (n/1140)
1	62	0.02	Central South University (China)	5.44%
2	46	0.08	Shanghai Jiao Tong University (China)	4.44%
3	29	0.03	Nanjing Medical University (China)	2.54%
4	29	0.05	Shandong University (China)	2.54%
5	28	0.05	Fudan University (China)	2.46%
6	27	0.05	Barbara Ann Karmanos Cancer Institute (USA)	2.37%
7	27	0.07	Sun Yat Sen University (China)	2.37%
8	25	0.06	Zhejiang University (China)	2.19%
9	24	0.09	Chinese Academy of Sciences (China)	2.11%
10	24	0.02	Wayne State University (USA)	2.11%

### 3.3 Analysis of references

The frequency of citation demonstrates the scholarly value of the article and the evolution of the research field. Among the 1140 documents retrieved, **Table 3** shows the top 10 most cited references, of which the document entitled “Copper induces cell death by targeting lipoylated TCA cycle proteins” by Tsvetkov et al. was cited most frequently (n=690). The first six of the ten articles are published in 2022. In addition, Tsvetkov P is included in two of the top 10 most cited papers, which is enough to show that he has a large influence in this field.

**Table 3:** Top 10 cited references related to cuproptosis in cancer research with citation.

Ranking	Count	Year	First author	Journal	Title	IF
1	690	2022	Tsvetkov P	SCIENCE	Copper induces cell death by targeting lipoylated TCA cycle proteins	54.5
2	250	2022	Ge EJ	NATURE REVIEWS CANCER	Connecting copper and cancer: from transition metal signaling to metalloplasia	80.3
3	178	2022	Tang DL	CELL RESEARCH	Cuproptosis: a copper-triggered modality of mitochondrial cell death	39.3
4	126	2022	Oliveri V	FRONTIERS IN MOLECULAR BIOSCIENCES	Selective Targeting of Cancer Cells by Copper Ionophores: An Overview	5.2
5	120	2022	Bian ZL	GENES	A Novel Cuproptosis-Related Prognostic Gene Signature and Validation of Differential Expression in Clear Cell Renal Cell Carcinoma	3.9
6	118	2022	Kahlson Martha A	SCIENCE	Copper-induced cell death Excess copper causes mitochondrial protein aggregation and triggers a distinct form of cell death	54.5
7	114	2019	Tsvetkov P	NATURE CHEMICAL BIOLOGY	Mitochondrial metabolism promotes adaptation to proteotoxic stress	15.6
8	102	2018	Jiang P	NATURE MEDICINE	Signatures of T cell dysfunction and exclusion predict cancer immunotherapy response	69.4
9	101	2021	Sung H	CA-A CANCER JOURNAL FOR CLINICIANS	Global cancer statistics 2020: GLOBOCAN estimates of incidence and mortality worldwide for 36 cancers in 185 countries	307.2
10	98	2020	Voli F	CANCER RESEARCH	Intratumoral Copper Modulates PD-L1 Expression and Influences Tumor Immune Evasion	13

### 3.4 Analysis of active journals

Total 1140 papers published in 346 journals. The goal of this analysis was to identify the journals having the greatest impact on the field, as indicated by high publication and cited counts. The top ten co-cited journals as shown in **Table 4**. The Impact Factor (IF), which is related to the number of times a journal is cited, is a widely used measure of a journal’s authority in specific fields (21). As we can see in **Table 4**, *CA: A Cancer Journal for Clinicians* has the highest IF among them, and it is ranked seventh (n=428) in terms of citation frequency. Furthermore, most cited journals are in Q1, among which Science has the highest number of citations(n=805).

**Table 4:** Top 10 co-cited journals in the field of cuproptosis in cancers.

Ranking	Count	Centrality	Cited Journals	IF	JCR
1	805	0.1	SCIENCE	54.4	Q1
2	559	0.04	CANCER RESEARCH	13	Q1
3	485	0.02	NATURE REVIEWS CANCER	80.3	Q1
4	475	0.03	NATURE	60.9	Q1
5	450	0.04	CELL	57.5	Q1
6	436	0.01	NATURE COMMUNICATIONS	17	Q1
7	428	0.03	CA-A CANCER JOURNAL FOR CLINICIANS	307.2	Q1
8	421	0.03	PROCEEDINGS OF THE NATIONAL ACADEMY OF SCIENCES OF THE UNITED STATES OF AMERICA	12	Q1
9	421	0.01	FRONTIERS IN IMMUNOLOGY	8	Q1
10	398	0.01	FRONTIERS IN ONCOLOGY	5.2	Q2

**Figure 6:**
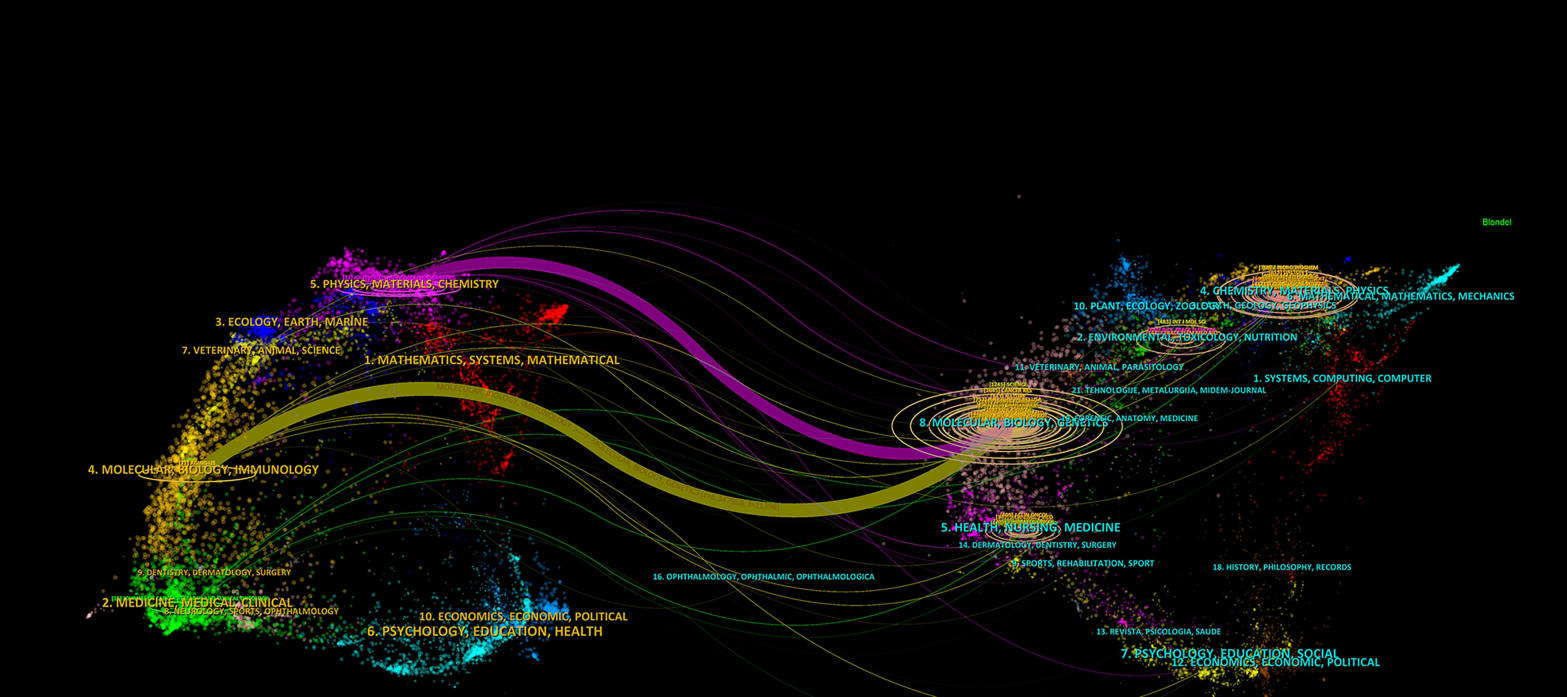
A dual-map overlay of journals related to cuproptosis in cancer therapy. The citing journals on the left and the cited journals on the right. The different colored paths indicate referential relationship.

CiteSpace software was utilized to create a dual-map overlay of the journals (**Figure 6**). The diagram illustrates the citing journals on the left and the cited journals on the right. It shows two paths, the orange and green paths indicate that papers published in ‘Molecular/Biology/Genetics’ journals were most cited in papers published in ‘Molecular/Biology/Immunology’ journals and ‘Medicine, Medical, Clinical’ journals.

### 3.5 Keywords analysis

**Figure 7:**
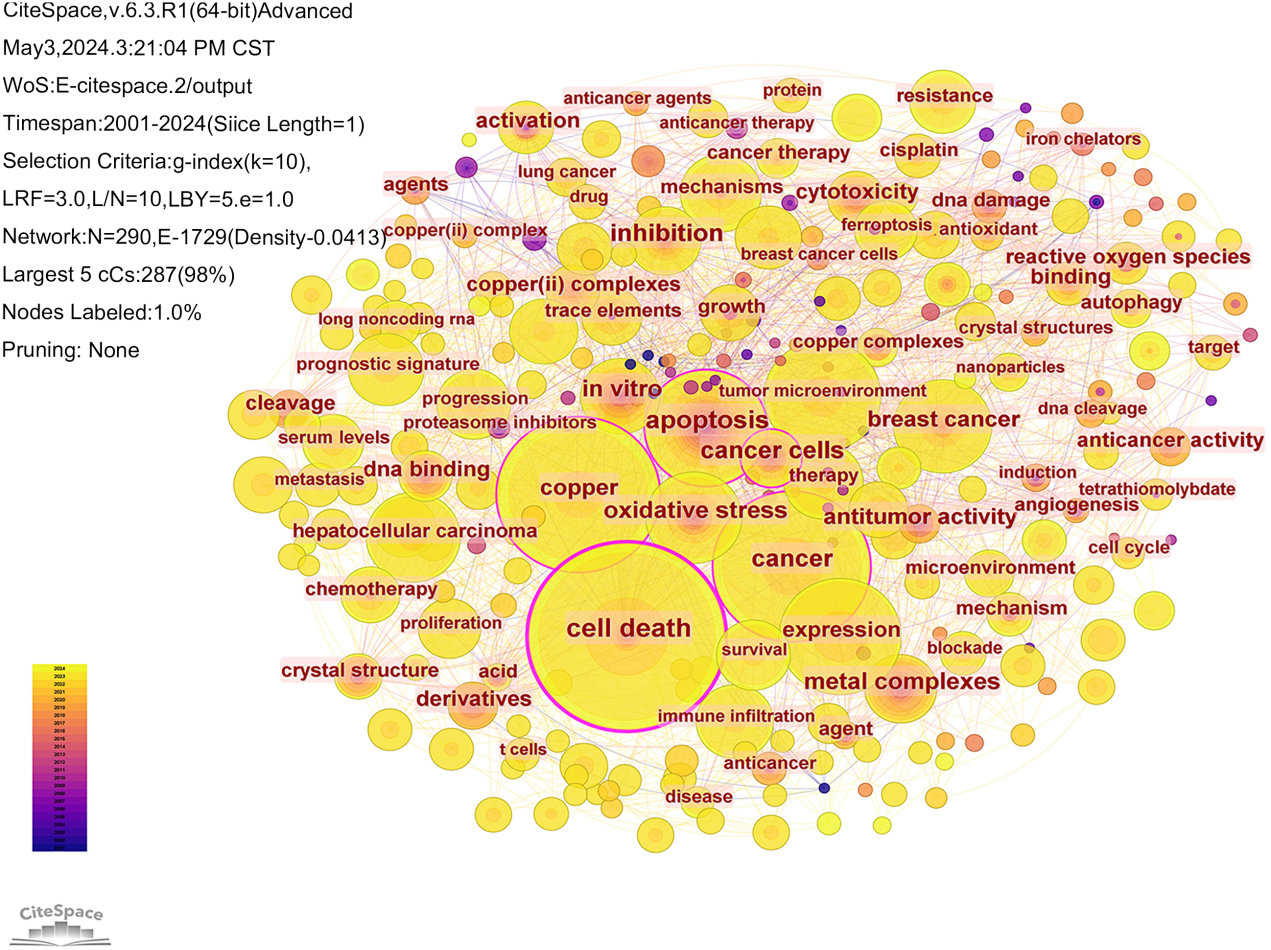
Collaboration analysis of keywords.

The literature was analyzed using CiteSpace software. A keyword co-occurrence map was generated, consisting of 290 nodes and 1729 links (**Figure 7**). The top 10 most frequently occurring keywords are listed in **Table 5**. Among them, the one with the highest frequency and centrality was “cell death” (n=360); in terms of cancer types, breast cancer and hepatocellular carcinoma had the highest frequency of occurrence, which is promising for antitumor therapy using cuproptosis as a cell death mechanism.

**Table 5:** Top 10 keywords in the field of cuproptosis in cancers in terms of frequency of occurrence.

Ranking	Count	Centrality	Year	Keywords
1	360	0.29	2005	cell death
2	245	0.11	2008	copper
3	237	0.15	2005	cancer
4	151	0.09	2007	expression
5	135	0.17	2006	apoptosis
6	116	0.04	2022	tumor microenvironment
7	99	0.09	2007	breast cancer
8	91	0.07	2010	oxidative stress
9	76	0.04	2019	hepatocellular carcinoma
10	67	0.09	2009	*in vitro*

**Figure 8:**
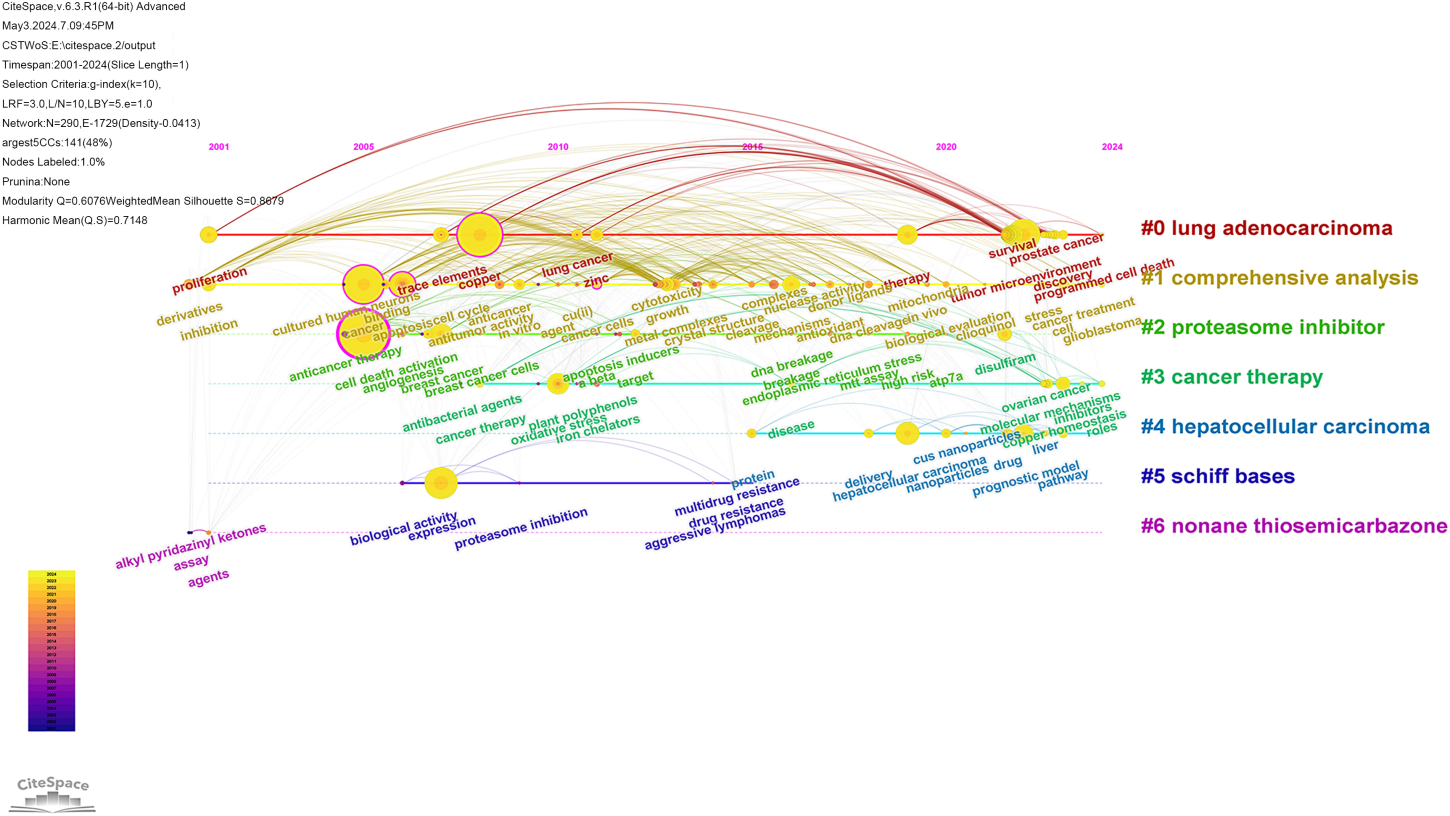
Timeline view of keyword related to cuproptosis in cancer therapy.

The CiteSpace software was used to visualize the timeline of the clustered keywords. As shown in **Figure 8**, the keywords are clustered, and as can be seen from the figure, the keywords were clustered into 7 clusters, including Cluster #0 lung adenocarcinoma, Cluster #1 comprehensive analysis, Cluster #2 proteasome inhibitor, Cluster #3 cancer therapy, Cluster #4 hepatocellular carcinoma, Cluster #5 schiff bases and Cluster #6 nonane thiosemicarbazone. Of the seven clustering tags, clusters #2, #5 and #6 relate to the mechanisms of cuproptosis in cancer research. Clusters #0 and #4 are hot cancer types studied in the area of cuproptosis. This visualization reflects the year of the appearance of each keyword, as well as its heat and duration. This allows us to understand the development history of each research direction and predict its future trends. The cluster of lung adenocarcinoma have lots of nodes that appeared around 2022 and have persisted until the present time. This suggests that scientists have been actively researching cuproptosis in the field of lung adenocarcinoma, which has promising future applications.

**Figure 9:**
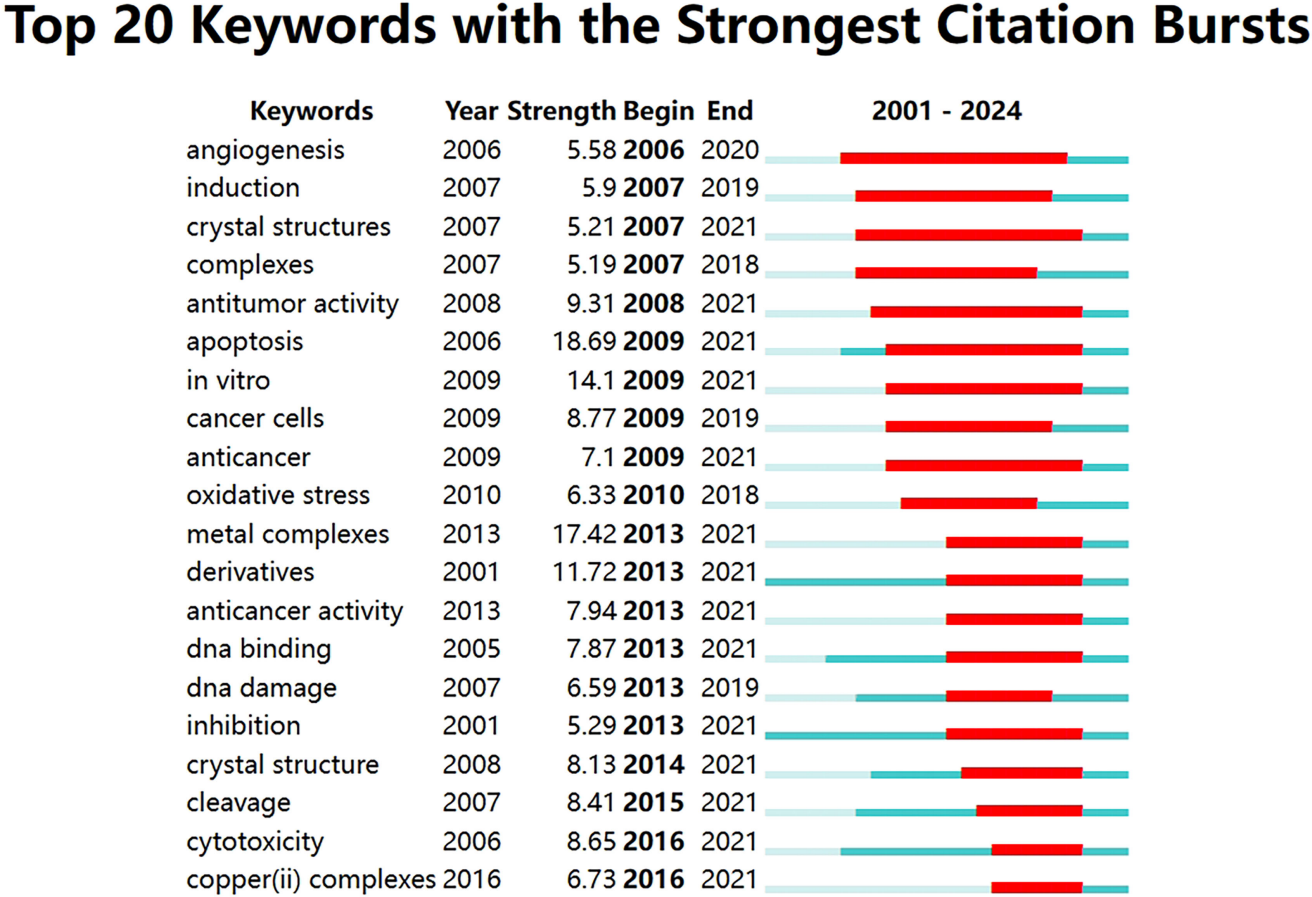
Top 20 keywords with the strongest citation bursts related to cuproptosis in cancer therapy.

The term ‘burst’ refers to a sudden and significant increase in keywords frequency over a period of time. Analysis of keywords bursts can indicate the development of cutting-edge topics in a field and provide some insight into future directions. The top 20 keywords with the strongest citation bursts were obtained by analyzing the keyword emergence of the 1140 papers included in the study. The red part representing the time period of the keyword explosion and the blue part represents the time interval, as shown in **Figure 9**. From 2009 to 2021, the term “apoptosis” had the highest citation burst strength, indicating that many studies compared cuproptosis with apoptosis to explore its mechanism. The keyword with the longest time span is “angiogenesis”, which lasts from 2006 to 2020. A study demonstrated that copper deficiency, whether induced by dietary restriction or by copper chelators, impairs a tumor’s capacity to elicit an angiogenic response (22). The keyword “antitumor activity” also has a long time span according to the figure. Antitumor activity refers to the ability of a drug to cause the growth or death of tumor cells by inhibiting the proliferation of tumor cells or the growth of tissue (23), which suggests that the development of antitumor drugs is one of the most important aspects of cuproptosis in antitumor therapy.

## 4 Discussion

In this paper, we discuss 1140 English-language publications in the WoSCC database as of 29 April 2024, which are highly relevant to the topic at hand. The literature included in this study comes from 63 countries, 3724 institutions, 6834 scholars, and 346 journals. A bibliometric study and visualization analysis of cuproptosis in cancers research was conducted, focusing on multiple aspects such as authors, countries, institutions, journals, references, and keywords. The study of cuproptosis began in the late 20th century when research was still in its early stages, with fewer and less mature publications; the development of this field remained stable until 2022 when Tsvetkov et al. formally proposed the concept of cuproptosis (8). Research on cuproptosis in the field of cancer has gained significant attention from scientists worldwide, leading to an explosion of studies in this emerging direction. Cuproptosis is predicted to remain a hot research topic in the future.

Although the number of articles in this field has continued to increase over the past two years, two concurrent problems need to be addressed. On the one hand, research on cuproptosis and cancer is in the developmental stage. Therefore, it is important to focus on innovative research and produce groundbreaking articles to inspire scholars’ ideas and drive further development. On the other hand, although Chinese scholars have published far more literature in this field than those from other countries (**Table 1**), the comprehensive impact and innovativeness of the articles need to be improved. Furthermore, China and the United States are the main hubs of cooperation, and global collaboration should be enhanced to advance the development of this area. It is noteworthy that China started its research in this field late, but has become one of the largest contributors in terms of output in recent years.

In the bibliometric analysis, keywords are the research theme and core content of the literature. Keyword co-occurrence analysis allows you to identify the distribution and growth of research topics within a given academic field. At the beginning of the study, the majority of keywords are associated with the molecular mechanism of cuproptosis (**Figure 9**), including reactive oxygen species (ROS) and oxidative stress (OS), among others. During the rapid development phase after 2022, most studies focused on genes and cellular levels, as well as related studies such as anti-tumor therapy. These studies offer new avenues for treating various types of cancer. Additionally, copper can have a direct or indirect impact on several cancer types, including breast, lung, and colorectal cancers, among others. It can also regulate the metabolic and signaling pathways of tumor cells (24). In addition, the keyword citation burst analysis showed that different emergent words appeared in each time period, indicating that there are many new thoughts and discoveries emerging from the research on cuproptosis in cancers, and these topics will also become hot research topics in recent years.

Research on cellular cuproptosis initially focused on experimental studies. The results of our bibliometric analysis demonstrate that the field of research on the relationship between cuproptosis and tumorigenesis is still in its infancy. The future will undoubtedly present significant challenges. While current research has elucidated the central role of aberrant copper metabolism in tumorigenesis, its therapeutic application remains hindered by multiple bottlenecks. Firstly, the complexity of copper metabolism and the incomplete elucidation of its molecular mechanisms pose significant barriers. The regulation of copper homeostasis is intricate, involving a network of epigenetic modulation and metabolic reprogramming. For instance, METTL16-mediated m6A modification stabilizes FDX1 mRNA, thereby enhancing cuproptosis sensitivity (25). However, the spatiotemporal synergy between epigenetic regulation and metabolic rewiring remains to be elucidated. Additionally, the intricate interplay between cuproptosis and ferroptosis, two distinct yet interconnected cell death modalities, remains to be fully elucidated. The interplay between these pathways may determine therapeutic windows, with potential synergistic or antagonistic effects requiring further investigation (26). Secondly, clinical therapies that rely on cuproptosis are confronted with limitations in drug delivery systems and inherent drug toxicity. Traditional copper ionophores, such as elesclomol, encounter challenges due to their limited water solubility and suboptimal targeting. Clinical trials have revealed that patients with high lactate dehydrogenase (LDH) levels exhibit reduced efficacy, a phenomenon that may be attributed to mitochondrial metabolic heterogeneity (27). While nanoparticle-based delivery systems have shown improvements in tumor penetration, challenges such as long-term toxicity and limited scalability in production persist. Thirdly, the tumor microenvironment (TME) is characterized by its profound complexity and dynamism. In the context of triple-negative breast cancer (TNBC), the TME manifests significant mechanical heterogeneity, marked by the presence of hyperactivated cancer-associated fibroblasts (CAFs) and aberrant ECM accumulation. This results in elevated tissue stiffness, increased solid stress, and compressed vasculature, exacerbating hypoxia. Hypoxic conditions activate the hypoxia-inducible factor (HIF) signaling pathway, suppressing FDX1 expression—a critical regulator of iron-sulfur cluster metabolism—and attenuating cuproptosis sensitivity (28).

It is imperative that future studies integrate multidimensional data and construct a precise typing system based on copper metabolic features. Additionally, these studies should explore the synergistic mechanism of copper death with immunotherapy and metabolic regulation. By doing so, we can promote its leapfrog development toward clinical application.

## 5 Hotspots and frontiers

Copper is regarded as a necessary trace element in the human body. It is also a cofactor of key enzymes such as cytochrome c oxidase, playing a vital role in the process of cellular respiration and energy production. In addition to serving as a cofactor of enzymes, copper also participates in regulating cell growth and function (29, 30). Insufficient copper intake can inhibit growth, while excessive intake may lead to oxidative stress, cell death, and tissue damage. Imbalance or deficiency of copper metabolism can cause harm to health and is related to various genetic diseases and metabolic dysfunction related diseases (31, 32). Therefore, maintaining a balanced intake of copper is crucial for maintaining human health.

Copper concentration in tumor tissue and blood of many cancer patients increased, including breast cancer, lung cancer and digestive tract cancer (20). Copper has a complex role in cancer, as it can promote tumor development and may also inhibit tumor growth. Copper is very important in the proliferation process of cancer cells, but excessive copper can contribute to the death of cells, especially through the mitochondrial pathway (30). With the improvement of the availability of copper ion carriers, the transport process of copper ions through the cell membrane is promoted, providing a new method for inducing cell death in anti-cancer treatment.

## 6 The mechanism of cuproptosis

In normal physiological conditions, the intracellular copper content is regulated by cells through a dynamic internal environmental balance mechanism, maintaining it level at a relatively low point. So as to prevent the occurrence of excessive copper accumulation and subsequent cell damage. The primary mechanism of copper toxicity is the intracellular accumulation of copper ions. Copper ions have been observed to directly bind to the acylation components in the TCA cycle, which has been demonstrated to result in the aggregation and dysregulation of these proteins. This process ultimately leads to the blocking of the TCA cycle, the induction of protein toxicity stress, and the subsequent triggering of cell death (8). In mammals, the small intestine efficiently absorbs copper from food. Copper ions are present in the extracellular space of the small intestine, occurring in the form of divalent copper ions. Prior to reduction to Cu (I) by binding to the metal reductase STEAP family, Cu (II) is unable to be directly absorbed by intestinal cells. Cu (I) primarily enters the intestine or other somatic cells via the SLC31 copper permeation family of transporters, specifically SLC31A1/CTR1 (solute carrier family 31mem-1) and SLC31A2/CTR2 (solute carrier family 31mem-2) (33).

In the cytoplasm, one of the copper chaperones of superoxide dismutase (CCS), such as SOD1, is mainly present in the cytoplasm, with a small portion in the mitochondrial intermembrane space. It is responsible for delivering copper to specific proteins, and its abnormal expression is closely associated with the growth and development of tumor cells (34). COX17 (cytochrome c oxidase copper chaperone 17) transports Cu (I) from the cytoplasm to mitochondrial membrane proteins SCO1 and SCO2 (cytochrome C-oxidase 1 and 2), and SCO2 introduces copper into MT-CO2/COX2 (mitochondrial encoded cytochrome C oxidase II) to facilitate the transport of copper ions to mitochondria. Another pathway of COX17 is by transferring copper to COX11 (cytochrome c oxidase copper partner COX11), which is then inserted into MT-CO1/COX1 (mitochondrial encoded cytochrome c oxidase I) (35). COX17 is a vital component in the functioning of MT-CO1 and MT-CO2 within the mitochondrial respiratory chain, and its role is pivotal to the growth, metastasis and invasion of tumors (36). Moreover, COX17 represents a promising molecular target for the treatment of solid and hematological malignancies (37).

The ATPase ATP7A and ATP7B, which transport copper, play a pivotal role in the extracellular secretion of copper ions (38). Under low copper levels, ATP7A and ATP7B are located in the reverse Golgi network. Copper is transported to the trans-Golgi apparatus via its copper partner, ATOX1 (antioxidant 1 copper partner). In the cytoplasm, ATOX1 binds to Cu (I) and transmits it to ATP7A and ATP7B in the Golgi network (39). As copper exposure increases, ATP7A and ATP7B are translocated to the plasma membrane or intracellular vesicles. Concurrently, ATP7A and ATP7B facilitate the transport of copper from the transitional Golgi network to the small vesicles situated on the opposite side of the Golgi apparatus. The copper-containing vesicles are capable of fusing with the plasma membrane, thereby releasing copper into the extracellular environment. Meanwhile, copper is transported to the nucleus through CCS, where it is able to activate the transcription factor HIF1 (hypoxia inducible factor 1) (40). In addition, ATOX1 can also transport copper to the nucleus, acting as a co-dependent transcription factor (39).

Cu^2+^ has been observed to bind to specific proteins within the mitochondria, thereby inducing toxic effects (41). Lipoylated mitochondrial enzymes, including DLAT, have been identified as primary targets of cuproptosis. The process of cuproptosis is initiated by the direct binding of copper to lipoylation sites, which results in the oligomerization of DLAT. This, in turn, leads to the disruption of the TCA cycle and subsequent protein toxicity. Furthermore, the inactivation of iron-sulfur (Fe-S) cluster assembly proteins (e.g., LIAS, FDX1) exacerbates mitochondrial dysfunction. FDX1, acting as a copper reductase, converts Cu^2+^ into the more toxic Cu^+^, which directly attacks Fe-S cluster proteins, leading to their disassembly and degradation (42). Gene knockout experiments demonstrate that depletion of FDX1 or LIAS significantly suppresses cuproptosis, confirming their central roles in this pathway (43).

Furthermore, lipoylation has been identified as an additional hallmark feature of cuproptosis. ACSL4 (Acyl-CoA synthetase long-chain family member 4) catalyzes the generation of acyl-CoA, promoting lipid peroxidation and ferroptosis (44). However, the role of lipoylation in cuproptosis is more specific. Studies reveal that copper ions cross-link lipoylated proteins to form irreversible polymers, resulting in mitochondrial membrane structural disruption and opening of the mitochondrial permeability transition pore (mPTP). This process differs from apoptosis or necrosis, as it does not depend on caspase activity or plasma membrane rupture but instead causes direct cell death through mechanical stress induced by protein aggregation. It is noteworthy that the inhibition of lipoylation effectively blocks cuproptosis, underscoring its pivotal role in the pathway (45).

It is evident that cuproptosis exhibits cross-regulation with other programmed cell deaths, including ferroptosis and pyroptosis. For instance, copper-induced lipid peroxidation shares molecular mechanisms with ferroptosis, yet cuproptosis possesses unique mitochondrial targets that define its independence (46). Furthermore, copper activates the NF-κB signaling pathway to induce inflammasome assembly, thereby triggering pyroptosis phenotypes (47). It is also notable that cuproptosis can synergize with immunogenic cell death (ICD). Treatment with copper ionophores such as elesclomol has been shown to upregulate calreticulin (CRT) exposure and HMGB1(high mobility group box 1 protein) release, thereby enhancing antitumor immune responses (48). These interconnections provide a theoretical basis for developing combination therapies.

## 7 Cuproptosis and cancers

**Figure 10:**
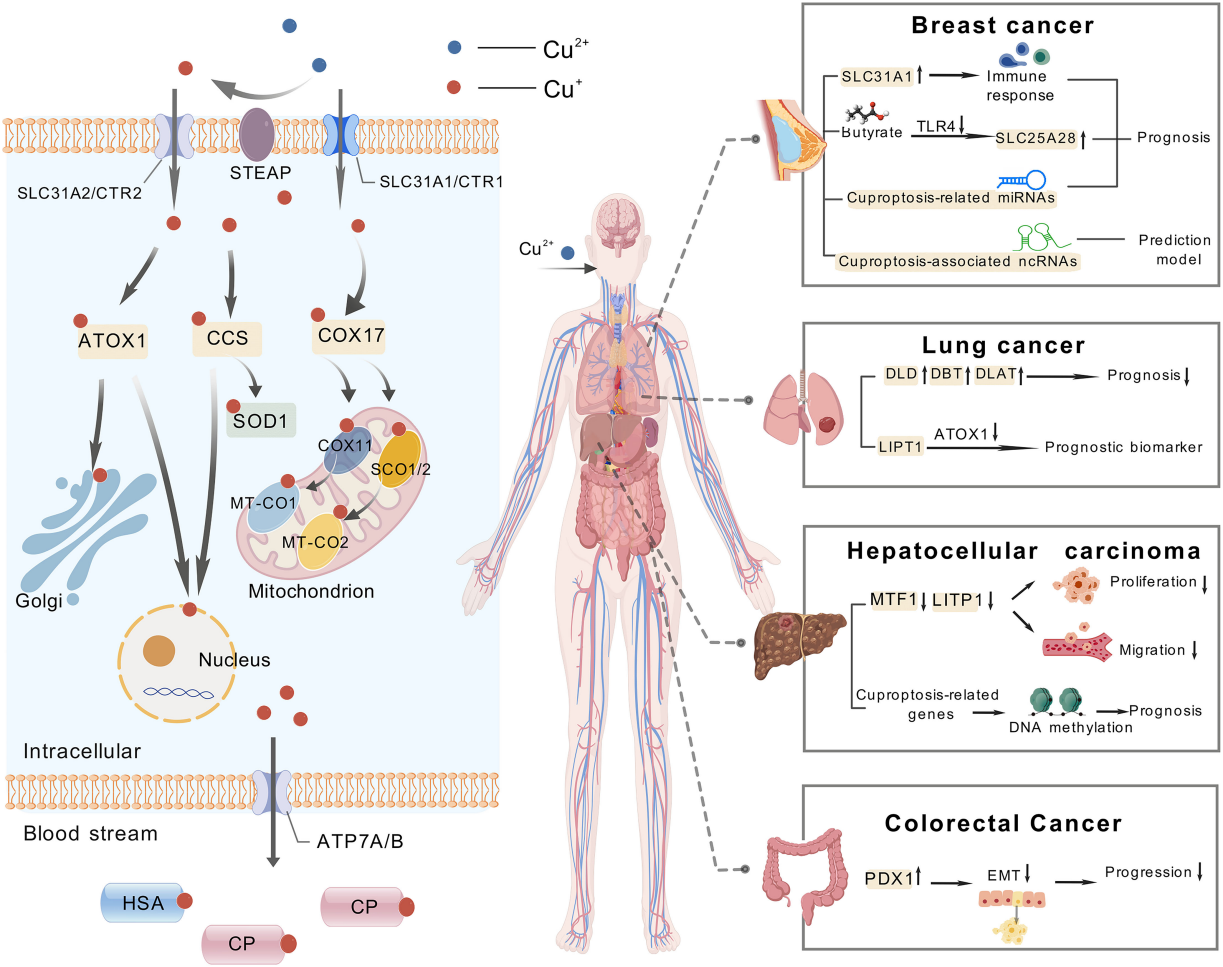
The mechanism of cuproptosis and examples of targeting cuproptosis in cancer treatment, including breast cancer, lung cancer, hepatocellular carcinoma and colorectal cancer.

A study of popular cancers in the cuproptosis research field was conducted using CiteSpace. The analysis revealed that breast cancer, lung cancer, hepatocellular carcinoma, and colorectal cancer have received the most research attention and have yielded the most results. Consequently, we mainly analyzed these four cancers. It is worth mentioning that **Figure 10** and **Table 6** summarize the cuproptosis-related mechanisms and related potential clinical therapeutic agents for these four cancers.

**Table 6:** A cuproptosis-based summary of potential therapeutic agents for four popular cancer types.

Cancer Types	Copper Ionophores	Copper Chelators	Copper-Based Nanomaterials
Breast cancer	DSF/Cu; ES	TM; ATN-224	DSF@PEG/Cu-HMSNs; Cu-GA NPs; CCDHs
Lung cancer	DSF/Cu; ES	TM; D-Penicillamine	CuO-NPs
Hepatocellular carcinoma	DSF/Cu; ES	TTM; Trientine	TCCHA
Colorectal cancer	DSF/Cu; ES	TM; Trientine; TPEN	Cu₂O@CaCO₃; Copper Nanoparticles

### 7.1 Cuproptosis and breast cancer

In a bioinformatics analysis, SLC31A1, a member of the copper transporter family, was selected as the most potential CRGs in breast cancer. In addition, there is a positive correlation between SLC31A1 and the presence of different degrees of immune cell infiltration, immune cell biomarkers, and immune checkpoint expression in breast cancer. The authors also found that high copy number degrees of SLC31A1 in cancer may induce its overexpression. Their data show that the cuproptosis-related gene SLC31A1 is a potential indicator to predict the diagnosis, prognosis and treatment response of breast cancer (49). In addition, another member of the family SLC25A28 (member 28 of the solute carrier family 25) also participates in butyric acid inhibiting the malignant biological behavior of breast cancer cells, which is achieved by butyric acid inhibiting TLR4 to promote the expression of SLC25A28 (50). Another study screened 5213 differentially expressed genes and 204 differentially expressed miRNAs related to cuproptosis between TNBC triple negative breast cancer and control samples, of which five miRNAs (miR-203a-3p, miR-1277-3p, miR-135b-5p, miR-200c-3p and miR-592) are closely related to the prognosis of triple negative breast cancer (51).

Numerous studies have shown that non coding RNA (ncRNA) has equally important biological functions as genes encoding proteins (52). In a study of predicting subtypes of breast cancer patients with cuproptosis-related ncRNAs, the author utilized the first five of the 94 ncRNAs that were greatly correlated with cuproptosis to construct a predictive model. This model revealed that these five ncRNAs exhibited differential expression in five distinct cancer subtypes and were associated with immune invasion, RNA modification, and angiogenesis. This result provides a new direction for accurate prediction of cancer subtypes and clinical treatment targets. With respect to the treatment of breast cancer, although the use of copper ion carriers has not yet become widespread in clinical practice, copper chelate mixture TM and AmphinexTM (ATN-224) have entered phase II clinical trials in the field of breast cancer (53). Within the domain of breast cancer treatment, copper ionophores enhance intracellular copper ion accumulation, inducing ​cuproptosis​ or synergizing with other mechanisms. ​ DSF​ forms a complex with copper, inhibits the ​Wnt signaling pathway, downregulating β-catenin and c-Myc expression, and concurrently activates ​ferroptosis​ by increasing lipid peroxidation and reducing ​GPX4/GSH levels, thereby promoting cell death in TNBC (26, 54)​. As a copper ionophore, elesclomol (ES) reduces Cu² ^+^ to the more toxic Cu ^+^ through binding with FDX1, promoting the oligomerization of lipoylated proteins within mitochondria and thereby triggering cuproptosis (7, 8). It has been demonstrated that certain copper-based nanomaterials exhibit clinical potential in the treatment of breast cancer. For instance, DSF@PEG/Cu-HMSNs—a nanocomposite composed of polyethylene glycol (PEG)-modified hollow mesoporous silica nanoparticles (HMSNs) loaded with disulfiram (DSF) and Cu² ^+^—achieves targeted delivery and sustained release through the nanoparticle’s structural properties. The underlying mechanism of cuproptosis involves the release of copper ions, which in turn leads to the abnormal aggregation of mitochondrial key proteins and the disruption of iron-sulfur cluster proteins. This series of events ultimately results in the execution of the cell through a process known as cuproptosis. Concurrently, DSF amplifies copper-induced cellular toxicity by inhibiting proteasome activity. In the 4T1 triple-negative breast cancer mouse model, the material demonstrated significant suppression of tumor growth while minimizing off-target tissue damage through HMSNs-mediated targeted delivery, exhibiting high efficacy and low toxicity with promising clinical translation potential (55).

### 7.2 Cuproptosis and lung cancer

The 13 core genes involved in cuproptosis in lung adenocarcinoma tissue were significantly elevated, with DLD, DBT (dihydrothiamine branched acylase E2), and DLAT (dihydrothiamine S-acetyltransferase) associated with poor prognosis. Cuproptosis affects the tumor microenvironment (TME) characteristics and heterogeneity of lung adenocarcinoma (56, 57). Using databases such as TCGA and CancerSEA, as well as single-cell sequencing, it was found that CRGs are mainly involved in copper ion transport, citric acid cycle (TCA cycle), and central carbon metabolism in lung adenocarcinoma cells. CRGs play a vital role in the regulation of DNA damage response, inflammation, and metastasis in lung adenocarcinoma, revealing that CRGs can serve as potential prognostic and immune biomarkers for lung adenocarcinoma (58). LIPT1 (fatty acid protein ligase 1) is also an important gene associated with cuproptosis and a central prognostic gene with reduced expression in non-small cell lung cancer (NSCLC). It inhibits the progression of copper chaperone gene antioxidant 1 (ATOX1) by inhibiting its expression, thus confirming that LIPT1 is a valuable prognostic biomarker in NSCLC (59). Disulfiram (DSF) can be used to treat NSCLC, and DSF greatly increases the expression of PD-L1 (programmed death ligand 1). Therefore, researchers have found that the combination of anti PD-L1 and DSF can enhance the inhibitory effect of cuproptosis on the activity of NSCLC cell A549 in non-small cell lung cancer (60). Additionally, Elesclomol (ES) has clinical applications in the treatment of lung cancer. By shuttling copper to mitochondria, ES induces oxidative stress and cuproptosis. Studies have demonstrated its ability to overcome cisplatin resistance in lung cancer cells (61). D-penicillamine, a copper chelator, has demonstrated selective toxicity toward LC cells and has been shown to enhance the lethality of radiation or carboplatin when administered concomitantly (62). In the clinical application of copper-based nanomaterials, studies have indicated that ​CuO-NPs (Copper Oxide Nanoparticles)​​ induce ​cuproptosis​ by generating ​ROS and disrupting mitochondrial function, achieving a ​54% inhibition rate against A549 cells (63).

### 7.3 Cuproptosis and hepatocellular carcinoma

The cuproptosis-related risk score (CRRS) was used to predict the prognosis of hepatocellular carcinoma (HCC) patients, and was validated in the GSE76427 and ICGC datasets. Patients with lower scores had better prognosis. In addition, the high grouping in HCC has identified three potential therapeutic targets (TUBA1B, CDC25B, and CSNK2A1) as well as a number of potential therapeutic compounds. Knocking down the prognosis cuproptosis-related gene MTF1 shows inhibition of proliferation, invasion, and migration in HCC cell lines (64). Following the construction of a survival curve for liver cancer patients using the TCGA database, it was determined that patients with low expression of the LIPT1 gene in liver cancer tissue exhibited a markedly improved prognosis. Additionally, the inhibition of LIPT1 gene expression was observed to impede the proliferation and migration of liver cancer cells, HepG2 and Hep3B (65). The model was developed using cuproptosis-related long non coding RNAs (lncRNAs) has shown good predictive ability for the prognosis and immunotherapy outcomes of hepatocellular carcinoma patients. The model demonstrates that the low-risk group exhibits a markedly longer survival period in comparison to the high-risk group (66). It has been demonstrated that alterations in DNA methylation can influence the incidence, progression, and management of a range of neoplastic diseases by modulating the transcription of pertinent genes. A recent study has revealed that cg05706061, a prognostic marker, is situated in the promoter region of the cuproptosis-related gene SLC31A2. There is a significant difference in the DNA methylation level of cg05706061 between liver cancer and normal tissues, and the expression of SLC31A2 is significantly correlated with this process (67). Most CRGs are upregulated in hepatocellular carcinoma, and overexpression of CRGs is related to low DNA methylation and low patient survival (68).

In the clinical treatment of HCC based on cuproptosis, Elesclomol (ES) and Disulfiram/Copper complex (DSF/Cu) also play critical roles. ES has been shown to induce cuproptosis by activating AMPK in liver and pancreatic cancer cells, thereby enhancing the cytotoxic effects of cisplatin in HCC when utilized in conjunction (8, 69). DSF (disulfiram) has been shown to chelate Cu² ^+^, forming an active complex that induces ICD and inhibits the PARP1/GSK3β signaling pathway. Furthermore, it has been observed that this process triggers the release of DAMPs in HCC, thereby enhancing anti-PD-1 efficacy, and promotes ferroptosis through GPX4 degradation (26, 70). The copper chelator Tetrathiomolybdate (TTM) has been shown to inhibit copper-dependent angiogenesis and tumor metastasis by chelating copper and reducing its bioavailability. Clinical studies have revealed the potential of Trientine to enhance the efficacy of cisplatin and inhibit angiogenesis in hepatocellular carcinoma. Trientine functions by targeting the copper-dependent enzyme LOX and blocking the IL-8-mediated angiogenic pathway. Trientine has been demonstrated to be efficacious in the inhibition of angiogenesis and the prevention of HCC development in rat model (71, 72). DSF chelates Cu² ^+^ to form an active complex, inducing ICD and inhibiting the PARP1/GSK3β signaling pathway. Furthermore, it has been observed that this process triggers the release of DAMPs in HCC, thereby enhancing anti-PD-1 efficacy, and promotes ferroptosis through GPX4 degradation. In recent research on copper-based nanomaterials, a study demonstrated that TCCHA, a multi-enzyme catalytic copper-based nanoplatform, generates hydroxyl radicals (·OH) via Cu² ^+^-mediated Fenton reactions to disrupt redox homeostasis by depleting glutathione (GSH). Simultaneously, it alleviates tumor hypoxia through catalytic decomposition of H _2_O _2_ into oxygen (O _2_), thereby enhancing the photodynamic effect mediated by chlorine e6 (Ce6)-produced singlet oxygen (¹O _2_). Loaded with the anti-angiogenic drug AL3818 to suppress tumor angiogenesis, TCCHA exhibits pH/GSH dual-responsive drug release properties. This enables synergistic chemodynamic/photodynamic/anti-angiogenic triple therapy, significantly inhibiting HCC cell proliferation, migration, and tumor growth without apparent systemic toxicity (73).

### 7.4 Cuproptosis and colorectal cancer

There is a positive correlation between CRGs and the key pathways associated with the initiation, progression, and metastasis of colorectal tumors. Research findings suggest that most CRGs are suppressed within tumors, correlating positively with SCNA (Somatic Copy Number Alterations) and negatively with DNA methylation. This further suggests that both SCNA and DNA methylation influence the expression of CRGs (74). Utilizing the TCGA database, it was discovered that the cuproptosis-related gene FDX1 is expressed less in colorectal cancer (CRC) tissues, and CRC patients with lower FDX1 expression experience poorer prognoses. Experimental validation has shown that FDX1 inhibits the growth and progression of CRC by suppressing the progression of EMT (75). Furthermore, the inverse relationship between cuproptosis and angiogenesis has been confirmed in colorectal cancer cell lines through the results of additional studies (76).

In the clinical treatment of colorectal cancer based on cuproptosis, copper ionophores represent a major research focus. Elesclomol (ES) transports Cu² ^+^ into cells via ES-Cu complex formation, inducing abnormal oligomerization of lipoylated proteins in the TCA cycle to trigger cuproptosis. ES induces ATP7A degradation, inhibits copper efflux, and promotes ROS accumulation (77). Disulfiram (DSF) forms a CuET complex with copper, suppresses aerobic glycolysis through the miR-16-5p and 15b-5p/ALDH1A3/PKM2 axes, activates ULK1 to induce autophagy, and promotes immunogenic cell death (78, 79). Among copper chelators, tetrathiomolybdate (TM) and trientine exhibit mechanisms in colorectal cancer consistent with their roles in other cancers, including inhibition of angiogenic factors and promotion of apoptosis in colorectal cancer cells. In addition, TPEN has been demonstrated to elicit HCT116 cell death through a process known as redox cycling and the generation of ROS. In the context of CRC cell lines, the combination of 5-fluorouracil (5-FU) and TPEN exhibited a synergistic effect in promoting apoptosis. This enhancement was attributed to an increase in reactive ROS production and Mcl-1 ubiquitination, resulting in augmented anticancer activity and a reversal of chemoresistance (80). In their seminal work, Chang et al. pioneered the development of release-based nanomedicine through the conceptualization of a core-shell nanostructure composed of Cu2O@CaCO3, a pioneering design that was further modified with hyaluronic acid. The objective of this design is to furnish synergistic therapies targeting colorectal cancer and initiated by the tumor microenvironment (TME), encompassing photothermal, photodynamic, chemo dynamic, and calcium overload-mediated methodologies (81).

## 8 Cuproptosis and cancer therapy

The field of nanotechnology has facilitated the clinical translation of cuproptosis through innovative material design, particularly demonstrating significant potential in cancer therapy. In addition to their role as effective drug carriers, they also deliver immediate therapeutic benefits. In view of the remarkable success of cisplatin in cancer treatment, copper chelators are extensively researched as potential alternatives. Moreover, nano-copper chelators are crucial copper-supplying agents. Integrating nanomaterials with copper chelators into a single system not only maintains therapeutic efficacy but also addresses the issues of high systemic toxicity, hydrophobic properties, and low bioavailability inherent to copper chelators, offering an alternative perspective and approach in cancer therapy (82).

Consequently, there is a prevailing emphasis on employing copper ion carriers and chelating agents to induce copper poisoning as a possible cancer treatment strategy. One approach involves providing copper-based agents to induce additional oxidative stress, while the other involves suppressing abnormal cell proliferation and potential metastasis through the depletion of endogenous copper (83). Future research is essential to perform an in-depth investigation into the role and mechanisms of copper cell death in tumors using various cell lines and animal tumor models, thereby establishing the foundation for considering CRGs as biomarkers and therapeutic targets in cancer diagnosis and treatment.

The employment of targeted delivery systems has been instrumental in enhancing the accumulation of copper ions within tumors. For instance, the Lipo-Ele@CuO _2_ nanocomposite facilitates the concurrent delivery of elesclomol and CuO _2_, exhibiting responsiveness to acidic tumor environments, resulting in the release of Cu² ^+^ and disulfiram (DSF). This process induces mitochondrial cuproptosis and enhances the sensitivity of radiotherapy. This approach has been shown to achieve tumor inhibition rates of 90.7% in lung cancer models and 97.3% in breast cancer models (84). The Au@MSN-Cu/PEG/DSF nanoplatform integrates photothermal effects with copper metabolism modulation, attaining 80.1% tumor inhibition in hepatocellular carcinoma (85). Furthermore, biomimetic nanorobots that have been engineered to mimic macrophage membranes have been developed to target tumors. These nanorobots release Cu2+ and TLR7/8 agonists to induce cuproptosis and secrete type I interferons, thereby activating systemic antitumor immunity. This strategy has been shown to have cross-organ anti-metastatic effects in mice (86). Another study developed a polymer, PHPM, which is sensitive to ROS. This polymer can encapsulate ES and copper into nanoparticles (NP@ESCu). Upon entering cancer cells, NP@ESCu releases ES and copper in response to excessive ROS, thereby inducing cuproptosis while reprogramming the immunosuppressive tumor microenvironment. It is important to note that NP@ESCu enhances immunotherapy efficacy in “cold tumors” when combined with αPD-L1 (87). In addition, a Cu-MOF nanoplatform integrating copper ions and photosensitisers combines photodynamic therapy (PDT) with cuproptosis, efficiently killing cancer cells and inhibiting angiogenesis, thereby reducing the risk of tumor recurrence in colorectal cancer models (88). A recent research development has been the design of a sandwich-like copper polyoxometalate nanocapsule (PWCu) featuring four ultrasmall CuO octahedra. This structure enables precise Cu^+^ release under radiotherapy radiation, thus overcoming acquired radioresistance by inducing cuproptosis (89).

Collectively, nanotechnology advances cuproptosis-based cancer therapy through multidimensional innovations, including targeted delivery, photothermal regulation, and immunomodulation, thus improving both precision and efficacy. Future efforts should focus on optimizing biocompatibility, *in vivo* stability, and scalable fabrication of nanocarriers, while exploring synergistic combinations with existing therapies to accelerate clinical translation.

## 9 Conclusion

Our bibliometric analysis of the literature on cuproptosis has revealed a burgeoning interest in this novel form of cell death, particularly in its applications to cancer therapy. The research landscape has witnessed a notable surge, with the United States and China emerging as frontrunners in this domain. Despite a later start, China has demonstrated a rapid ascent in the field of cuproptosis research. Initially, investigations centered on the molecular underpinnings of cuproptosis, with a particular focus on its interplay with other forms of regulated cell death, such as ferroptosis and apoptosis. Presently, the research trajectory has shifted towards elucidating the role of cuproptosis in various cancers and the potential for therapeutic intervention.

In essence, this study not only captures the current state of cuproptosis research but also lays the foundation for future endeavors aimed at harnessing the power of cuproptosis to combat cancer. The findings highlight the potential for cuproptosis to revolutionize our understanding of tumor biology and to pave the way for more effective and targeted cancer therapies.

## Data Availability

The original contributions presented in the study are included in the article/supplementary material. Further inquiries can be directed to the corresponding author.
